# Profile: The Kenya Multi-Site Serosurveillance (KEMIS) collaboration

**DOI:** 10.12688/gatesopenres.15569.2

**Published:** 2025-02-27

**Authors:** E. Wangeci Kagucia, Shirine Voller, Abdhalah K. Ziraba, Godfrey Bigogo, Patrick K. Munywoki, Kimani Makobu, D. James Nokes, James Nyagwange, Cameline Orlendo, Donald Akech, Antipa Sigilai, Clayton Onyango, Bonventure Juma, Amy Herman-Roloff, Peninah Munyua, Caroline Apondi, Shirley Lidechi, Allan Audi, Alice Ouma, George Aol, Thomas Misore, Caroline Nasimiyu, Dickens Onyango, Terrence Lo, Kadondi Kasera, Rose Jalang'o, Leonard Kingwara, Ifedayo Adetifa, Anthony O. Etyang, George Warimwe, Ambrose Agweyu, J. Anthony G. Scott

**Affiliations:** 1 KEMRI-Wellcome Trust Research Programme, Kilifi, Kenya; 2 Nuffield Department of Medicine, University of Oxford, Oxford, UK; 3 London School of Hygiene and Tropical Medicine, London, UK; 4 African Population and Health Research Center, Nairobi, Nairobi County, Kenya; 5 Kenya Medical Research Institute Centre for Global Health Research, Kisumu, Kenya; 6 United States Centers for Disease Control and Prevention, Nairobi, Kenya; 7 Zeeman Institute for Systems Biology and Infectious Disease Epidemiology Research (SBIDER) and School of Life Sciences, University of Warwick, Warwick, UK; 8 Kenya Medical Research Institute Centre for Global Health Research, Nairobi, Kenya; 9 Washington State University Global Health Kenya, Nairobi, Kenya; 10 Paul G Allen School of Global Health, Washington State University, Pullman, Washington, USA; 11 County Department of Health, Kisumu, Kenya; 12 Ministry of Health, Government of Kenya, Nairobi, Kenya; 13 Nigeria Centre for Disease Control and Prevention, Abuja, Nigeria

**Keywords:** HDSS, multiplex, integrated serosurveillance, SARS-CoV-2, COVID-19, partnership, Kenya, national serosurveillance platform

## Abstract

The Kenya Multi Site Serosurveillance (KEMIS) collaboration set out to implement an integrated, nationally representative, population-based program of serological surveillance for past infection for a number of important infectious diseases in Kenya. The project started in December 2021 and built on a portfolio of SARS-CoV-2 research conducted in 2020 and 2021. In this profile paper, we describe the background of the KEMIS collaboration, its aim and objectives, the Health and Demographic Surveillance System sites that were involved in data collection, and the key activities undertaken. We also explain how we established governance and management of the KEMIS collaboration, and reflect on opportunities, challenges, lessons learned, and future directions.

## Background

Monitoring the burden of disease is challenging in settings such as Kenya where national health records data collection systems are constrained. In such situations, serosurveillance using population surveys to define the prevalence of specific antibodies to infectious diseases can offer a valuable approach for outbreak characterization and monitoring and evaluation of disease prevention and control programs. 

Thus, in 2020, serosurveillance was central to the approach taken by a group of research institutions in Kenya, the United States, and the UK that came together to respond to a call from the Government of Kenya for technical support to manage the COVID-19 pandemic in Kenya. These institutions were: the KEMRI-Wellcome Trust Research Programme (KWTRP), the Africa Population Health and Research Center (APHRC), the Kenya Medical Research Institute Centre for Global Health Research (KEMRI-CGHR), the US Centers for Disease Control and Prevention (US CDC), the London School of Hygiene and Tropical Medicine (LSHTM) and the University of Warwick. These institutions collaborated to deliver an integrated program of demographic, clinical and laboratory surveillance. The Bill & Melinda Gates Foundation (BMGF), US CDC and UK Foreign, Commonwealth and Development Office (FCDO) provided funding for the program of serosurveillance in various rural and urban parts of Kenya. This program of research ended in December 2021 and its results have been published ^1– 9^. 

Generating evidence to inform national policy in Kenya was a key driver for the first program of COVID-19 research, and links with the Government of Kenya were strengthened. This work positioned the research partners well to respond to a subsequent call from BMGF for serosurveillance that would extend beyond SARS-CoV-2 to include other pathogens of national interest. Together, the partners crafted a proposal to implement an integrated, nationally representative, population-based serosurveillance program in Kenya while continuing to conduct SARS-CoV-2 serosurveillance. The Kenya Multi-Site Serosurveillance (KEMIS) collaboration was launched on 1 December 2021 with the aim of generating representative seroprevalence data from five Health and Demographic Surveillance System (HDSS) sites during 2022 and 2023.

## KEMIS collaboration objectives

The specific objectives of the proposed work were:

1. To track SARS-CoV-2 antibody seroprevalence in 2022 and 2023 at five HDSS sites in Kenya, including antibody kinetics among individuals followed up longitudinally in a subset of HDSS sites.
2. To integrate seroprevalence data into transmission dynamic models to forecast disease patterns.
3. To produce policy relevant output (e.g., disease burden under specified vaccine scenarios) and recommendations for decision makers.
4. In collaboration with the Kenya Ministry of Health, to identify priority epidemic pathogens (e.g., Ebola, Lassa fever, Marburg, Congo-Crimean Haemorrhagic Fever, Middle East Respiratory Syndrome, Zika) for public health serosurveillance.
5. To extend serosurveillance to indicator epidemic diseases (Rift Valley Fever [RVF], chikungunya, dengue, and any newly emerging pathogens), as a demonstration project for integrated serosurveillance.
6. To develop multiplex assays on the Luminex platform for priority epidemic, endemic and vaccine-preventable disease (VPD) pathogens as identified through engagement with MoH.
7. To develop a framework for future integrated nationally representative serosurveillance in collaboration with other stakeholders (e.g., National AIDS and STIs Control Program [NASCOP], National Malaria Control Program [NMCP], Kenya National Bureau of Statistics [KNBS]).
8. To conduct annual review meetings with a broad base of stakeholders (e.g., disease control program managers, local/ global policymakers, national/ sub-national governments, and funders) where we will: share output from 2021–23 serosurveillance activities, solicit feedback on policy outputs and/or share progress towards development of the integrated nationally representative serosurveillance platform.

## The KEMIS HDSS sites

The HDSS sites participating in KEMIS comprised the Kilifi HDSS, Nairobi Urban HDSS, Manyatta HDSS, Kibera Population-Based Infectious Disease Surveillance (PBIDS) and Asembo PBIDS. The Kilifi HDSS, Nairobi Urban HDSS, Kibera PBIDS, and Asembo PBIDS sites have been previously profiled ^10– 14^. Characteristics of each of the five sites participating in the KEMIS collaboration are included in Table 1.

**Table 1.** Characteristics of Health and Demographic Surveillance System sites.

HDSS Site (Lead organization)	Year established	Geographic region	Rural/ urban	Population (census month & year)	Administrative county (% of national population) *
**Asembo PBIDS** **(KEMRI-CGHR/ CDC-Kenya)**	2006	Western	Rural	35,219 (Apr 2022)	Siaya (2%)
**Manyatta HDSS** **(KEMRI-CGHR/ Kisumu** **County)**	2016	Western	Urban	70,502 (Jun 2022)	Kisumu (2%)
**Kibera PBIDS** **(KEMRI-CGHR/ CDC-Kenya)**	2006	Nairobi	Urban	22,235 (Apr 2022)	Nairobi (9%)
**Nairobi Urban HDSS** **(APHRC)**	2002	Nairobi	Urban	90,000 (Oct 2021)	Nairobi (9%)
**Kilifi HDSS** **(KWTRP)**	2000	Coast	Rural	308,581 (Apr 2022)	Kilifi (3%)

*Percent of the total population resident within the counties is calculated using the national 2019 census data ^15^.

**Figure 1.**
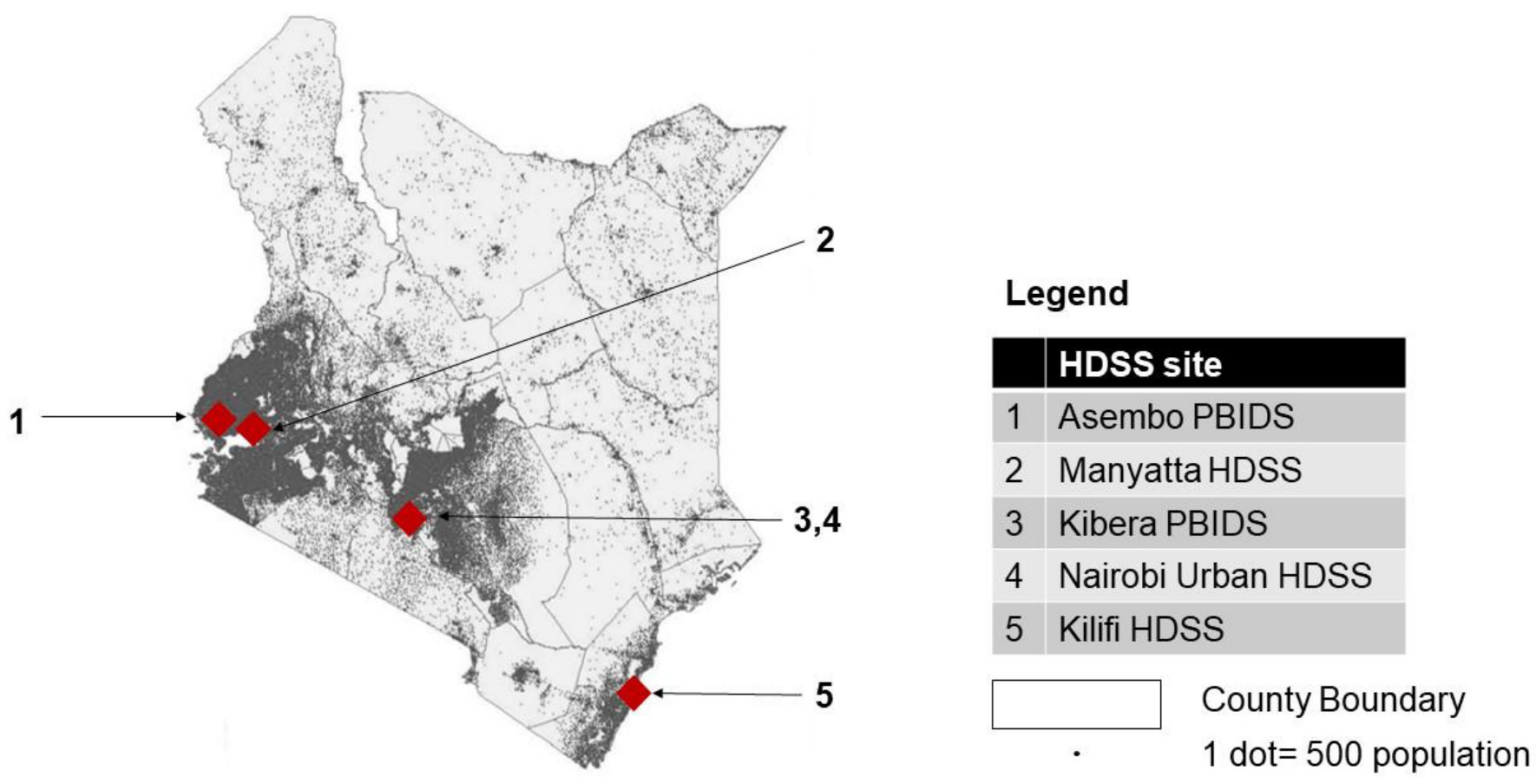
Geographical location of KEMIS HDSS sites.

The HDSS sites were located in the western (Manyatta HDSS and Asembo PBIDS), south-eastern (Kilifi HDSS), and central (Kibera PBIDS and Nairobi Urban HDSS) parts of Kenya. The population resident within the counties in which the HDSS sites were located comprised 17% of Kenya’s total population. Though located in the southern half of the country, the sites represented the densely populated regions of Kenya ( Figure 1 and Table 1). They were also representative of the typology of premature disease-related mortality in Kenya, represented by clusters of high or low mortality, though they excluded clusters of high mortality in sparsely-populated northern Kenya ^16^. The sites represented both urban (three sites) and rural (two sites) Kenya ( Figure 1 and Table 1).

## Key activities: Serosurveys, SARS CoV2 antibody testing, assay development, modelling, and stakeholder engagement

### Serosurveys

In 2022, one survey each at the Kibera PBIDS and Asembo sites was conducted, and two surveys each at the Kilifi HDSS, Nairobi Urban HDSS ^17^, and Manyatta HDSS sites. One survey at each site was conducted in 2023.

The Kilifi and Nairobi HDSS sites applied a repeated cross-sectional survey design. Using the respective HDSS site’s census as a sampling frame, a randomly selected, age-stratified sample of 850 individuals were selected. The sample included 100 children in each five-year age band <14 years of age, 50 individuals in each five-year age band between 15 and 64 years of age, and 50 adults aged ≥65 years. This sample size was sufficient to measure seroprevalence of 50% with precision (95% confidence interval [CI]) of 47–53%. The Manyatta HDSS, Kibera PBIDS and Asembo PBIDS sites used a longitudinal survey design. A random sample of 225 households, targeting a total of 900 members in the sampled households, was selected from each site’s respective population register. This sample size was sufficient to measure seroprevalence of 50% with an associated 95% CI of ±5% assuming a design effect of 2.

Trained field staff visited selected participants at the households and sought written informed consent for participation. Consent was obtained from the guardians of children aged <18 years; children aged 13–17 years provided written assent as per local ethical guidelines. Approximately 2mL of venous blood were collected from participating children <5 years of age and approximately 5mL from individuals aged ≥5 years. Blood samples were collected by study-employed phlebotomists either at home or at a nearby health facility using either heparinized or serum separator tubes. They were then processed to separate out plasma or serum at a central laboratory. The plasma or serum samples were then biobanked at -80°C for future testing.

Ethical approval to conduct the serosurveys was obtained from the Kenya Medical Research Institute Scientific and Ethics Review Unit (4085, 4168), the Oxford Tropical Research Ethics Committee (44-20), and the London School of Hygiene and Tropical Medicine Research Ethics Committee (26950). Kibera, Asembo, and Manyatta serosurveys were also reviewed by US CDC and were conducted consistent with applicable federal law and CDC policy as provided for in the Code of Federal Regulations (45 C.F.R part 46 and 21 C.F.R. part 56).

### SARS-CoV-2 antibody testing

Samples were tested for anti-SARS-CoV-2 immunoglobulin G (IgG) antibodies using validated assays. Samples collected at the Kilifi HDSS and Nairobi HDSS sites were tested centrally at the KWTRP laboratories. Those samples were tested for anti-spike IgG using the KWTRP anti-S IgG ELISA with 93% sensitivity (95% CI 88-96%) and 99% specificity (95% CI 98-99%). Development and validation of the assay has been previously described ^1^. Samples were considered positive for anti-S IgG when the ratio of the sample optical density (OD) over the negative control OD was >2. In addition to qualitative (positive/ negative) anti-S IgG results, binding antibody concentrations were also calculated by calibrating the anti-S IgG ELISA optical densities against the WHO International Standard ^18^. Samples collected during the initial round of 2022 surveys at the Kilifi HDSS and Nairobi HDSS sites were also tested for anti-nucleoprotein IgG using the KWTRP anti-N ELISA with 83% (95% CI 76-88%) sensitivity and 91% (95% CI 86-95%) specificity.

Samples collected at the Kibera PBIDS site were tested for anti-S IgG at the CDC-supported KEMRI-CGHR laboratories in Nairobi using the SCoV-2 Detect™ IgG ELISA kit (InBios International, Inc.), whose performance was similar to that of the KWTRP ELISA, with 92% sensitivity and 99% specificity ^19^. Samples collected at the Asembo PBIDS and Manyatta HDSS sites were tested for anti-S IgG using the InBios IgG ELISA kit at the CDC-supported KEMRI-CGHR laboratories in Kisumu.

As of December 2022, SARS-CoV-2 serosurveillance revealed sustained temporal increases in cumulative SARS-CoV-2 infection with rural-urban heterogeneity in seroprevalence ^17^. The latter finding underscores the need for targeted COVID-19 vaccination strategies, given limited public health resources, such as the prioritization of vulnerable groups residing in rural settings. Ongoing SARS-CoV-2 serosurveillance will be important for informing our understanding of age-specific population immunity dynamics, including waning, and the implications for the national COVID-19 vaccination strategy ^20^. 

### Multiplex assay optimization and development

To demonstrate the feasibility of integrated serosurveillance and to support the rational use of samples, we proposed to leverage existing assays at KWTRP for optimization or development of multiplex assays. We prioritized the optimization/ development of assays that would support the generation of policy-relevant outputs (Objective #5 of the collaboration). With the support of the Netherlands National Institute for Public Health and the Environment (RIVM), KWTRP investigators had previously implemented a multiplex assay testing for IgG antibodies to seven vaccine preventable diseases (VPDs), i.e., diphtheria, pertussis, tetanus, measles, mumps, rubella and varicella. In addition, the development of an arbovirus multiplex assay began in 2023. Optimization of the seven-plex VPD assay under KEMIS was pursued as VPD serosurveillance can inform priorities for disease-specific immunization programs while arbovirus serosurveillance can inform pandemic preparedness efforts. These applications of serosurveillance can be achieved through identification of population immunity gaps, prediction of outbreak risk, and prediction of the impact of disease control and prevention interventions ^21– 23^. For example, the generation of age-specific measles serological profiles as part of KEMIS can inform refinement of national vaccine deployment policies including targeted supplementary immunization activities and optimization of the infant immunization schedule.

The RIVM VPD multiplex assays have been validated and described in detail previously ^24, 25^. These VPD assays were initially optimized for implementation at KWTRP on MAGPIX, a multiplex bead-based fluorescence immuno-assay. We sought to further optimize the seven-plex VPD assay to be run on a different flow cytometry multiplex platform, the Luminex 200. Briefly, commercial antigens (diphtheria toxoid, pertussis toxin, tetanus toxoid, measles virus, mumps virus, rubella virus, and varicella zoster virus) were conjugated to carboxylated polystyrene or magnetic microspheres following standard operating procedures. Successful conjugation was confirmed by assessing the mean fluorescence intensity (MFI) of a reporter conjugated to a secondary antibody bound to the primary antibody of a known sample. Conjugation was validated by assessing correlation after testing a panel of samples with known concentration provided by the RIVM (i.e., validation panel); assessing the linearity and recovery of calibration curves using established standards; and evaluating variation in the concentration of controls as measured in different runs. Samples were run in duplicate dilutions (1:200 and 1:4000) on the Luminex 200 and plates were read using the Luminex xPONENT ^®^ software. Antibody concentrations were interpolated from MFI adjusted for background fluorescence using five-parameter logistic regression-fit standard curves generated in Belysa software (Sigma). Samples with >20% intra-assay variation were run again. During optimization of the seven-plex assay, we used the same IgG antibody positivity cut-offs used by RIVM for diphtheria, tetanus, measles, mumps, rubella and varicella; these cut-offs were supported by prior evidence ( Table 2) ^26– 31^. An arbitrary cut-off of 5 IU/mL was used for optimization of the pertussis component of the seven-plex VPD assay; there is no acknowledged correlate of protection of pertussis, though IgG antibody levels ≥20 IU/mL are thought to indicate recent vaccination or infection ^32, 33^. 

**Table 2.** Pathogen-specific IgG antibody cut-offs for the seven-plex vaccine-preventable disease multiplex assay.

Pathogen	Cutoff
Diphtheria	0.01 IU/mL
Tetanus	0.01 IU/mL
Measles	0.12 IU/mL
Mumps	45 AU/mL
Rubella	10.0 IU/mL
Varicella	0.26 IU/mL

The antigens to be included in the multiplex arbovirus assay will be informed by arbovirus recombinant protein ELISAs existing or under development at KWTRP for Rift Valley Fever (glycoprotein [Gn] and C (Gc) antigens) ^34^, dengue (non-structural protein 1 [NS1] antigen and envelope [E] protein) and chikungunya (envelope protein 1 [E1] and 2 [E2] antigens). Assay-specific validation will be performed using gold standard negative and positive panels based on PCR or neutralization titers. The assay-specific cut-offs for seropositivity will be designed to maximize specificity. Antigen conjugation will be conducted at KWTRP and conjugation validation performed following a similar approach as for the VPD assay.

Development of multiplex assays for other pathogens will be contingent on funding and the outcome of an exercise on the identification of priority pathogens for serosurveillance, described elsewhere in this article. One aliquot of samples from all other sites is planned to be shipped to Kilifi for testing of antibodies to other pathogens, as agreed upon by the KEMIS collaborators.

### Modelling

Over the first two years of the pandemic in Kenya, a series of mathematical models were developed by a team from KEMRI-Wellcome Trust Research Programme and the University of Warwick Zeeman Institute to support the Kenya Government’s emergency response to COVID-19. This resulted in an enhanced understanding of the underlying epidemiology ^35^, predictions of disease incidence from introduced variants of concern ^36^ and forecasts of the potential impact of non-pharmaceutical interventions and vaccines on cases of disease, hospitalisation, and deaths ^37^. This work was underpinned by key data sets, including national PCR and lateral flow SARS-CoV-2 testing data, genomic surveillance of variants, and serosurveys at various time points from locations across the country. The serosurveys, funded through BMGF and FCDO grants as described in the introduction, defined the extent of population transmission of SARS-CoV-2, fundamental to model calibration ^35^.

Modelling work for KEMIS continues this evidence synthesis for COVID-19, to capture the dynamics of the virus as it is likely to move towards endemicity. The availability of age and spatial stratified seroprevalence data has enabled the exploration of the potential impact of age and geographical targeting of COVID-19 vaccines and boosters, forecasting the potential impact of future introduction of variants into the population, and the effects of both naturally acquired and vaccine acquired immune decay on disease risk and burden.

The broadened range of infectious disease serosurveys will enable modelling that aims to increase understanding of the epidemiology of vaccine preventable childhood diseases and vector-borne viruses. For example, in the case of mumps, KEMIS will produce the first cross-sectional antibody surveys in Kenya which will inform on the pristine (i.e. in the absence of vaccination) age-stratified rates of transmission of this virus. A compartmental transmission dynamic model incorporating this information will provide predictions of the impact of adding mumps containing vaccine to the current national 2-dose measles-rubella containing vaccine strategy, assessing the potential impact on meningitis and orchitis disease.

### Identification of priority pathogens for serosurveillance

An extensive stakeholder engagement exercise was designed to generate a list of priority pathogens for serosurveillance in Kenya (Objective #4 of the collaboration). The study team engaged purposively selected stakeholders from the Kenya Ministry of Health, county departments of health, global and non-governmental organisations involved in disease surveillance, as well as academic and research organisations in developing the list of priority pathogens. A modified Delphi technique was used, involving three stages of stakeholder engagement. At the first engagement, key informants were invited to an in-depth interview to identify a ranked list of priority pathogens for serosurveillance. After the interview, respondents were given an opportunity to refine their recommendations via email – the second point of engagement. The third engagement involved a consensus building workshop where participants representing the stakeholders described above developed a ranked, consensus list of priority pathogens for serosurveillance. The outcome of the consensus building workshop has been described in a Policy Brief ^38^ and a scientific publication describing the entire qualitative exercise is underway.

## Project governance, management, and communication

From the outset, the KEMIS collaboration partners agreed that regular, transparent communication and a clear governance structure were essential for the smooth delivery of the complex, integrated program of work. The coordinating institution was KWTRP. A steering committee was established, with representation from each of the partner institutions and regional and national Ministry of Health. A representative of the funder, BMGF, was invited to attend as an observer. The steering committee met quarterly to review project progress and make strategic decisions. Committee documentation was saved in Microsoft Teams and made accessible to all steering committee members.

**Figure 2.**
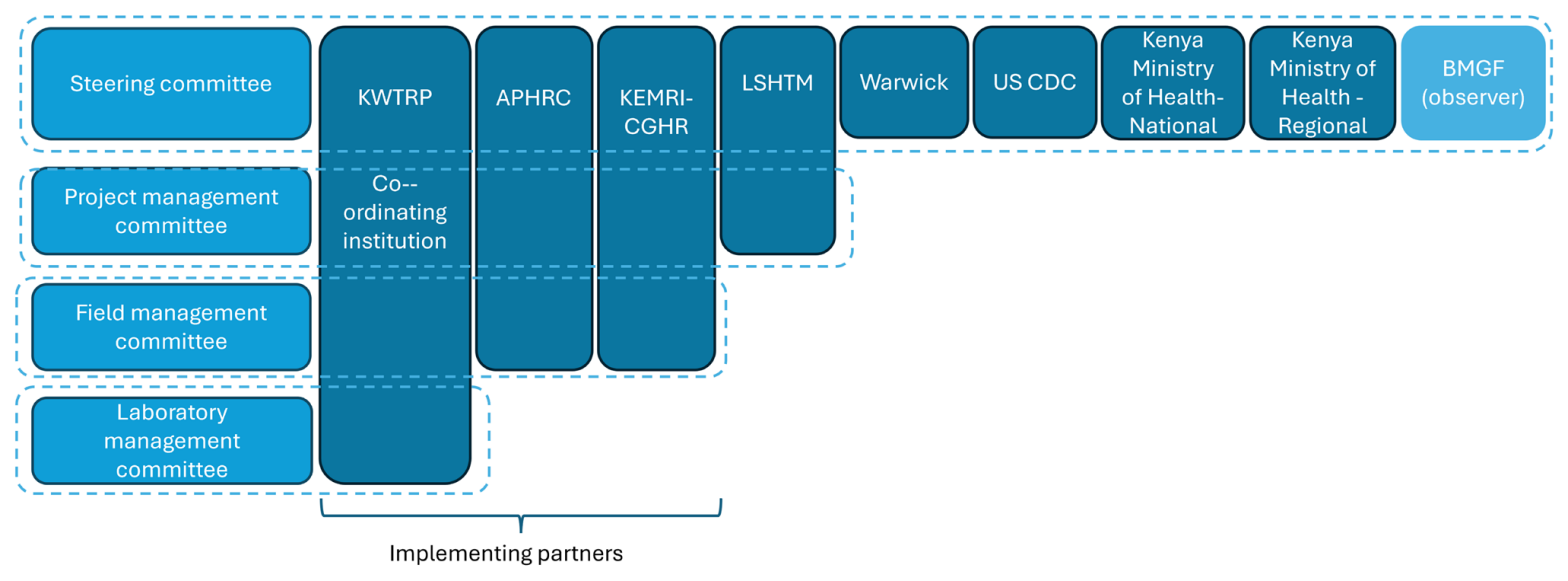
KEMIS project management and governance.

Separate committees were established to oversee project management, field management, and laboratory management. Figure 2 illustrates membership of the various management and governance structures for KEMIS.

Implementation of serosurveys at each of the five HDSS sites was the responsibility of the partner institution running that site, as identified in Table 1. above. However, decisions about the frequency and timing of surveys and survey design were discussed together and agreed upon by the steering committee to ensure cohesion and to facilitate analysis. 

In addition to KEMIS-specific meetings, partners attended quarterly meetings organised by BMGF. During these meetings, progress updates and results from the other BMGF serosurveillance investments in Malawi and South Africa were shared and issues affecting the overall strategic direction of the portfolio of investments were discussed.

## Data management, ownership and communication of results

Partners agreed that the data from each HDSS site remains under the ownership of the institution managing that site. A minimum set of agreed-upon key variables (e.g., age, sex, date of sample collection, etc.) was collected as part of serosurveys at each site.

Partners had a strong commitment to sharing data in as close to real-time as possible through scientific publications ^39^ and policy briefs ^40^ and actively pursued opportunities to present to the Kenya Ministry of Health, WHO regional groups and the funder ^20, 41, 42^. In addition to the presentation of preliminary results, partners remain committed to the timely writing-up of results for publication and discuss the collaboration’s dissemination strategy in quarterly steering committee meetings. Data have been availed alongside the associated publications in line with institutions’ data access and sharing policies. Serosurveillance data were uploaded on a rolling basis on a KWTRP-supported epidemiological data dashboard ^43^.

## Opportunities, challenges and lessons learned

The decline in the incidence of severe COVID-19 disease and the resulting shift in focus from emergency response to endemic disease control ^44^ during 2022 and 2023 provided both an opportunity and a challenge. The opportunity was that declining interest in SARS-CoV-2 pushed the project team to accelerate the development of the laboratory assays and surveillance platform for the identification of other pathogens of interest. The challenge was in determining the level of effort to continue to invest in SARS-CoV-2 serosurveillance given continuing uncertainty regarding potential emergence of new variants of concern, longevity of protection from vaccines and natural exposure, and the implications on the scale and impact of future waves of infection.

Working across five sites through three implementing partners required careful planning, regular communication and respect between partners to strike an appropriate balance between consistency of approach and site-specific adaptation. The establishment of sub-committees described above allowed for detailed discussion at the relevant level without overburdening all project team members. Meanwhile, having an overall project management committee and steering committee worked well to manage coordination across project activities.

## Future directions

The KEMIS collaboration was intended to generate high quality data about SARS-CoV-2 and other infectious diseases through an integrated, population-based programme of serosurveillance, meanwhile demonstrating proof of concept for the establishment of a long-term, national platform for serosurveillance of priority pathogens in Kenya. 

Potential future directions include the continued development and validation of assays for detecting pathogens of interest, integrating HDSS and laboratory data into pathogen-specific epidemiological models, and developing new platforms to further strengthen national representativeness (Objective #7 of the collaboration). Approaches to extend representativeness and sustainability could include leveraging existing sample collection platforms, such as blood donors, and partnering with other disease surveillance initiatives such as national household malaria and HIV surveys. It will remain essential to continue to engage with the Ministry of Health, county governments, regional partners including WHO AFRO and Africa CDC and other key stakeholders to ensure that evidence generated has the potential to be used to inform national policy decisions.

## Conclusion

We describe the successful implementation of a nationally-representative population-based integrated serosurveillance platform in Kenya. To date, the collaboration has generated evidence to inform COVID-19 prevention and control, with generation of seroprevalence data for other key pathogens underway. It has established robust inter-institutional relationships and an operational structure that could be used for future surveillance initiatives. Our experience may inform other similar efforts in sub-Saharan Africa. 

## Disclaimer

The views expressed in this article are those of the authors. Publication in Gates Open Research does not imply endorsement by the Gates Foundation or represent the official position of the U.S. Centers for Disease Control and Prevention.

## Data Availability

No data are associated with this article.
