# Supplementary material for: Profile: The Kenya Multi-Site Serosurveillance (KEMIS) collaboration
Source: Gates Open Res. 2025 Feb 27;8:60. Originally published 2024 Jun 18. [Version 2] doi: 10.12688/gatesopenres.15569.2 (PMC12149407; doi:10.12688/gatesopenres.15569.2)
Supplement: Peer review history

# Reviewer report: Reviewer response for version 2

**DOI:** 10.21956/gatesopenres.17710.r39640

**Published:** 2025-07-03

**Authors:** Pablo Martinez De Salazar

**Affiliations:** 1 Swiss Tropical and Public Health Institute, Alschwil, Switzerland

**This perspective paper describes a multi-site population-based surveillance network in Kenya designed to monitor infectious diseases of public health importance, including epidemic-prone pathogens (arboviruses, hemorrhagic fevers) and vaccine-preventable diseases. The network leverages lessons learned from a successful SARS-CoV-2 seroprevalence initiative.**

**The paper addresses an important topic by detailing the surveillance framework's objectives, structure, and planned activities. It has potential value as a model for similar initiatives in other countries and settings.**

**This reviewer finds that the paper would benefit from improved organization and clarity in several areas:**

**Content balance and focus: The SARS-CoV-2 serosurvey details are disproportionately extensive, particularly technical aspects like antibody testing protocols, as it is the description of the multiplex method. The authors could streamline these sections while emphasizing methodological elements that generalize to future surveys, such as randomization strategies and sample size requirements for diseases with lower prevalence and circulation (particularly relevant for early monitoring of emerging pathogens).**

**Pathogen selection clarity: The distinction between pathogens identified as surveillance priorities versus those actually included in current multiplex assays remains difficult to follow. A summary table categorizing targeted pathogens by implementation status would improve comprehension.**

**Timeline and implementation phases: The objectives could be clearly categorized by implementation status and expected outputs—what has been completed, what is currently underway, and what is planned for the future.**

**Public health impact: The paper lacks a clear description of how the network's outputs will inform public health decision-making beyond generating prior estimates for epidemiological models. A dedicated section outlining expected public health applications and research contributions would strengthen the manuscript.**

**Community engagement: While governance and data management are well-described, the framework for community engagement and community stakeholders involvement requires elaboration. How are community perspectives incorporated into surveillance priorities and implementation?**

**To enhance clarity and impact, the authors could: reduce SARS-CoV-2 (and multiplex) technical details while emphasizing transferable methodological principles; provide clear description for pathogen panel selection; explicitly describe expected public health outputs, if there are beyond informing epidemiological models; and detail community engagement strategies.**

Does the article adequately reference differing views and opinions?

Yes

Are all factual statements correct, and are statements and arguments made adequately supported by citations?

Yes

Is the Open Letter written in accessible language?

Partly

Where applicable, are recommendations and next steps explained clearly for others to follow?

Partly

Is the rationale for the Open Letter provided in sufficient detail?

Yes

Reviewer Expertise:

Infectious diseases epidemiology, epidemiological modeling

I confirm that I have read this submission and believe that I have an appropriate level of expertise to confirm that it is of an acceptable scientific standard, however I have significant reservations, as outlined above.

# Reviewer report: Reviewer response for version 2

**DOI:** 10.21956/gatesopenres.17710.r39475

**Published:** 2025-06-09

**Authors:** Mairead G Whelan

**Affiliations:** 1 University of Calgary, Calgary, Canada

Thanks for the opportunity to review this open letter describing the KEMIS serosurveillance program in Kenya, a collaborative initiative to serologically survey populations in Kenya for antibodies against COVID-19 and other viruses. I commend the authors for their thorough description of the program, methodological rigour, and accomplishments in executing this work over the course of the pandemic. The paper is scientifically sound but could be further improved from some small changes.

 I have a few minor suggestions and points of clarity that I suggest elaborating on. 

 1. The authors outline the aims and purpose of the paper itself in the abstract, but it would be ideal to add one to three lines in the main body, setting out the outline and purpose of the paper, and justification for this profile. This is why I answered 'partly' to the first question, and it would be easily remediated. 

 2. Where possible, it would be good to be specific as to sampling dates and dates of participant follow up in the methods and elsewhere. 

 3. On the HDSS sites: How and why were those sites selected in the first place? 

 4. In key activities and methods/information about the population: Please describe any information on vaccination efforts undertaken in these populations at the times of sampling/research. Where was sampling timed in relation to COVID-19 peaks/variants? How often was follow-up for the longitudinal cohort? 

 5. Antibody testing: Who were the assays validated by? Briefly mention whether performance was assessed by the manufacturer, an independent third party, the author team in-house, or other. For samples tested with multiple assays, do you have validation measurements for the combined assay series? Were the assays/results able to differentiate between vaccine-induced antibodies and natural infection acquired antibodies, and how were these results considered (if relevant?) 

 6. In the pathogen prioritization exercise: How were community member perspectives/priorities included in the interviews and exercise? 

 7. Data management and results sharing: How fast was this data sharing able to be accomplished, and do you have any insights on how the network may have improved on this compared to others? For context, Donnici et al. (citation attached) measured a 154-day median delay between serological sampling and first available reporting of results during COVID-19, which you may wish to use to compare to your group's comparative timeliness. 

 8. On that note, I would be curious to have a bit more discussion on the challenges/roadblocks identified in establishing KEMIS, so others can learn from the practical challenges and possible solutions offered here especially from an LMIC perspective. 

 9. Future directions: Has KEMIS considered exercising the upcoming WHO Unity pan-respiratory pathogen network/protocols? More of a curiosity than a suggestion. 

 The letter is informative and details a successful collaborative initiative in serosurveillance during the COVID-19 pandemic, and its transition beyond into multi-functional disease surveillance.

Does the article adequately reference differing views and opinions?

Yes

Are all factual statements correct, and are statements and arguments made adequately supported by citations?

Yes

Is the Open Letter written in accessible language?

Yes

Where applicable, are recommendations and next steps explained clearly for others to follow?

Yes

Is the rationale for the Open Letter provided in sufficient detail?

Partly

Reviewer Expertise:

Seroepidemiology, serosurveillance data standardization, evidence synthesis, infectious diseases, dashboard science

I confirm that I have read this submission and believe that I have an appropriate level of expertise to confirm that it is of an acceptable scientific standard.

## References

[1] : Timeliness of reporting of SARS-CoV-2 seroprevalence results and their utility for infectious disease surveillance. *Epidemics* .2022;41: 10.1016/j.epidem.2022.100645 10.1016/j.epidem.2022.100645 PMC958362436308993

# Reviewer report: Reviewer response for version 1

**DOI:** 10.21956/gatesopenres.16925.r38034

**Published:** 2024-10-14

**Authors:** Moses Masika

**Affiliations:** 1 University of Nairobi, Nairobi, Nairobi County, Kenya

This open letter describes the Kenya Multi Site Serosurveillance (KEMIS), a population-based program of serological surveillance for past infection for several infectious diseases in Kenya. The authors describe the aims of KEMIS, the approach and key outputs since the work commenced in 2021.

 In my view, the authors have described the KEMIS program clearly and demonstrated its importance and expected outcomes.

 I suggest a revision for the following two areas:

1. For the last paragraph on page 5 which describes the MAGPIX assay as a *‘fluorescent flow cytometry multiplex platform,’* I would suggest a change to ‘multiplex bead-based fluorescence immuno-assay’ as the assay does not really focus on cytometry by on protein targets.
2. On Project governance, management, and communication, consider including a flow diagram to illustrate the operations of KEMIS

The open letter describes a surveillance program that is well suited for the current challenges and resources available in Kenya and other similar countries. It will make for an informative read for persons engaged in disease surveillance and research.

Does the article adequately reference differing views and opinions?

Yes

Are all factual statements correct, and are statements and arguments made adequately supported by citations?

Yes

Is the Open Letter written in accessible language?

Yes

Where applicable, are recommendations and next steps explained clearly for others to follow?

Yes

Is the rationale for the Open Letter provided in sufficient detail?

Yes

Reviewer Expertise:

Infectious Diseases, Emerging Infections, Surveillance and Diagnostics, One Health and Antimicrobial resistance

I confirm that I have read this submission and believe that I have an appropriate level of expertise to confirm that it is of an acceptable scientific standard.

# Response

**Published:** 2025-02-14

**Authors:** Shirine Voller

**Affiliations:** Not Applicable, London, UK

Many thanks to Dr. Masika for the comments.  We agree that the text describing the MAGPIX assay should be amended to "multiplex bead-based fluorescence immuno-assay".

 We have developed a schematic to provide a visual depiction of the project governance and management structures and would be happy for this to be added to the paper.
